# Application of through-time spiral GRAPPA to phase velocity mapping (PVM)

**DOI:** 10.1186/1532-429X-16-S1-W20

**Published:** 2014-01-16

**Authors:** Robin Simpson, Jennifer Keegan, Nicole Seiberlich, David Firmin

**Affiliations:** 1 CMR Royal Brompton Hospital, Imperial College, London, London, UK; 2 Dept of Biomedical Engineering, Case Western Reserve University, Cleveland, Ohio, USA

## Background

PVM is an established technique for measuring blood [1] and myocardial velocities [2]. However, long scan times can restrict its application. Peak-GRAPPA has been used to accelerate Cartesian PVM up to a factor of 6 (R = 6) without degrading peak velocity measurements [3], however use of efficient k-space trajectories could allow higher temporal resolution (TR) in similar scan time. Through-time spiral GRAPPA allows highly accelerated spiral data to be reconstructed using through-time calibration information [4]. By collecting multiple repetitions of fully sampled calibration data, which can be collected without gating during free breathing, through-time information can be used to generate GRAPPA weights specific to the local undersampling in the non-Cartesian data. This abstract presents preliminary work on a single volunteer to apply this reconstruction to myocardial spiral PVM data.

## Methods

Data for phase velocity mapping were collected on a 3T Skyra MRI scanner (Siemens Medical Solutions, Erlangen, Germany) with 18 receiver channels. Fully-sampled k-space was made up of eight spiral interleaves, each of 14 ms duration, with acquired spatial resolution of 1.7 × 1.7 mm. Calibration data were collected (without ECG gating) by acquiring all eight spirals sequentially 30 times without velocity encoding. PVM data, including velocity compensated data and three directions of encoded data, were undersampled by a factor of two (four out of the eight interleaves) and acquired during a 16 heartbeat breath-hold with TR of 24 ms. Through time spiral GRAPPA reconstructions were performed at R = 2, and on data retrospectively undersampled to R = 4 (two spiral interleaves) and R = 8 (one interleaf). The reconstructed images were analysed and compared to a CG-SENSE [5] R = 2 reconstruction of the same data without background correction.

## Results

**Figure 1:**
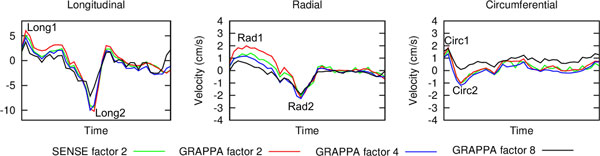
**Global longitudinal, radial and circumferential velocity-time curves measured for SENSE factor 2 and GRAPPA factors 2, 4 and 8**. The main peaks in each direction are labelled. Global peak velocities measured from these curves can be found in Table 1.

Figure 1 shows global longitudinal, radial and circumferential peak velocities measured using images generated by the four reconstructions (through-time spiral GRAPPA R = 2, R = 4, and R = 8, and CG SENSE R = 2), while Table 1 shows measured global peak velocities. The curves have very similar shapes in all cases. All peak velocities are well preserved up to R = 4, and some are also well preserved at R = 8 (Long1, Circ1, Rad2). However some of the peaks (Long2, Rad1, Circ2) are beginning to be degraded by R8.

**Table 1:** Global peak velocities measured with the various reconstructions

Peak	SENSE R2 (cm/s)	GRAPPA R2 (cm/s)	GRAPPA R4 (cm/s)	GRAPPA R8 (cm/s)
Long1	5.08	6.03	4.64	3.73

Long2	-9.40	-10.2	-9.97	-7.16

Rad1	1.40	1.99	1.16	0.78

Rad2	-2.03	-2.19	-2.26	-1.92

Circ1	1.63	1.84	1.34	1.78

Circ2	-1.04	-1.01	-1.19	0.09

## Conclusions

Through-time spiral GRAPPA can be used to accelerate spiral PVM acquisitions. In this example very little degradation of peak velocities was seen up to R4 and results at higher factors could be improved with further optimisation. Spiral trajectories have allowed higher TR than was possible in a similar breath-hold duration with peak-GRAPPA accelerated Cartesian tissue PVM acquisitions [3] (24 ms vs 70 ms). This technique shows great potential for the acceleration of spiral PVM.

## Funding

Robin Simpson is funded by HRUK grant RG2584.
